# Evaluation of Biocompatibility, Anti-Inflammatory, and Antinociceptive Activities of Pequi Oil-Based Nanoemulsions in In Vitro and In Vivo Models

**DOI:** 10.3390/nano12234260

**Published:** 2022-11-30

**Authors:** Andréia C. Pinheiro, Alicia S. Ombredane, Willie O. Pinheiro, Laise R. Andrade, Vitória R. P. Silva, Gisela J. Felice, Débora S. Alves, Aryanne F. Albernaz, Ariane P. Silveira, Milena C. F. Lima, Valdir F. Veiga-Junior, Thamis F. S. Gomes, Emanuel A. M. Damasceno, Fabiane H. Veiga-Souza, Paulo E. N. Souza, Sônia N. Báo, Eliza C. B. Duarte, Marcella L. B. Carneiro, Ricardo B. Azevedo, Mani I. Funez, Graziella A. Joanitti

**Affiliations:** 1 Laboratory of Bioactive Compounds and Nanobiotechnology (LBCNano), Campus Universitário—Centro Metropolitano, University of Brasilia, Ceilândia Sul, Brasília 72220-275, DF, Brazil; 2 Post-Graduation Program in Nanoscience and Nanobiotechnology, Institute of Biological Sciences, Campus Universitário Darcy Ribeiro, University of Brasilia, Brasília 70910-900, DF, Brazil; 3 Postgraduate Program in Health Sciences and Technologies, School of Ceilândia, Campus Universitário—Centro Metropolitano, University of Brasilia, Ceilândia Sul, Brasília 72220-275, DF, Brazil; 4 Laboratory of Protein Chemistry and Biochemistry, Department of Cell Biology, Institute of Biological Sciences, Campus Universitário Darcy Ribeiro, University of Brasilia, Brasília 70910-900, DF, Brazil; 5 Laboratory of Microscopy and Microanalysis, Department of Cellular Biology, Institute of Biological Sciences, Campus Universitário Darcy Ribeiro, University of Brasilia, Brasília 70910-900, DF, Brazil; 6 Chemistry Section, Military Institute of Engineering, Praça Gen. Tibúrcio, 80, Praia Vermelha, Rio de Janeiro 22290-270, RJ, Brazil; 7 Health Department, Nucleus of Cytopathology and Anatomic Pathology, Regional Hospital of Taguatinga, Taguatinga, Brasilia 72120-970, DF, Brazil; 8 Pharmaceutical Sciences School, Faculty of Ceilândia, Campus Universitário—Centro Metropolitano, University of Brasilia, Ceilândia Sul, Brasília 72220-275, DF, Brazil; 9 Laboratory of Electron Paramagnetic Resonance, Institute of Physics, Campus Universitário Darcy Ribeiro, University of Brasília, Brasília 70910-900, DF, Brazil; 10 Department of Pathology, Faculty of Medicine, Campus Universitário Darcy Ribeiro, University of Brasilia, Brasília 70910-900, DF, Brazil; 11 Post-Graduation Program in Biomedical Engineering—PPGEB, Faculty of Gama—FGA, University of Brasilia, St. Leste Projeção A–Gama Leste, Brasília 72444-240, DF, Brazil; 12 Nursing Course, School of Ceilândia, Campus Universitário—Centro Metropolitano, University of Brasilia, Ceilândia Sul, Brasília 72220-275, DF, Brazil

**Keywords:** nanoemulsion, pequi oil, anti-inflammatory, nanobiotechnology, bioactive compounds, antinociceptive, *Caryocar brasiliense*, biocompatibility, drug delivery system

## Abstract

Pequi oil (*Caryocar brasiliense*) contains bioactive compounds capable of modulating the inflammatory process; however, its hydrophobic characteristic limits its therapeutic use. The encapsulation of pequi oil in nanoemulsions can improve its biodistribution and promote its immunomodulatory effects. Thus, the objective of the present study was to formulate pequi oil-based nanoemulsions (PeNE) to evaluate their biocompatibility, anti-inflammatory, and antinociceptive effects in in vitro (macrophages—J774.16) and in vivo (*Rattus novergicus*) models. PeNE were biocompatible, showed no cytotoxic and genotoxic effects and no changes in body weight, biochemistry, or histology of treated animals at all concentrations tested (90–360 µg/mL for 24 h, in vitro; 100–400 mg/kg p.o. 15 days, in vivo). It was possible to observe antinociceptive effects in a dose-dependent manner in the animals treated with PeNE, with a reduction of 27 and 40% in the doses of 100 and 400 mg/kg of PeNE, respectively (*p* < 0.05); however, the treatment with PeNE did not induce edema reduction in animals with carrageenan-induced edema. Thus, the promising results of this study point to the use of free and nanostructured pequi oil as a possible future approach to a preventive/therapeutic complementary treatment alongside existing conventional therapies for analgesia.

## 1. Introduction

Inflammation is a physiological process characterized by the orchestration of different components of the immune system forming a defense mechanism against pathogens and tissue injuries that promotes the return of tissue to its homeostasis [1]. The development of this process is triggered by the dynamics of cellular events, coordinated by pro- and anti-inflammatory signals; however, the hyperactivation of these mechanisms results in chronic inflammatory responses, autoimmunity, and tissue damage, which play a critical role in the pathogenesis of several diseases [2].

Plants are relevant sources of molecules that can regulate the inflammatory process and can be used in the treatment of inflammatory diseases [3]. Advances in bioprospection, based on traditional uses, have led to the development of synthetic drugs widely used in clinical practice, such as acetylsalicylic acid, the active ingredient of the most commercialized anti-inflammatory in the world, first isolated from the bark of trees of the Salicaceae family [4].

Pequi oil is extracted from the pulp of the fruit produced by the *Caryocar brasiliense* tree, which is widespread in the northeast, mid-west, and north of Brazil. In traditional medicine, this oil is widely used in the treatment of respiratory infections, asthma, skin irritation, and wound-healing diseases that share an inefficient or exacerbated inflammatory process in their origin [5,6]. The therapeutic properties of this oil are related to the presence of molecules with biological activity, such as oleic acid, the predominant compound in the oil’s composition and which can inhibit the production of pro-inflammatory mediators of the NF-κB pathway through antagonism in toll-like receptor 4 [7].

Despite the relevant anti-inflammatory activity of pequi oil, its composition (rich in fatty acids) limits its therapeutic use, since these molecules in contact with aqueous biological fluids form agglomerates of low dispersion, which results in low biodistribution of the oil [6]. Nanotechnology provides tools to circumvent such pharmacokinetic limitations through drug-delivery systems [8,9,10].

Nanoemulsions are a type of lipid nanocarrier formed by the colloidal dispersion of immiscible liquids mediated by a surfactant, an amphipathic substance that works as a bridge that reduces the surface tension that exists between liquids and results in the formation of droplets of nanometer size [11,12,13]. The chemical structural organization of the nanodroplets protects the oil from oxidative and enzymatic degradation, and the nanometric size promotes greater surface contact with the cells, thus optimizing the pharmacokinetics of the oil and boosting its biological effects [8].

Several studies have described the anti-inflammatory and antinociceptive activities of free pequi oil in different models [5,6,14,15,16,17]. Interestingly, to the best of our knowledge, only one study has evaluated the anti-inflammatory effects of a nanostructured pequi oil in an in vivo model [6]. Therefore, considering the advantages of using lipid nanocarriers for hydrophobic samples and the fact that little information regarding the anti-inflammatory and antinociceptive effects of daily oral treatment with pequi oil nanoemulsion (PeNE) is available, the aim of the present study was to formulate and characterize PeNE in order to investigate its anti-inflammatory and antinociceptive effects, along with its biocompatibility in in vitro and in vivo models.

## 2. Materials and Methods

### 2.1. Materials

Pequi oil was donated by Farmacotécnica (a pharmacotechnical development company, Brasilia, Brazil). It was extracted from the fruit pulp by cold pressing and filtration. Egg lecithin was purchased from Lipoid (Ludwigshafen, Germany). MTT (3-[4,5-dimethylthiazol-2-yl]-2,5-diphenyltetrazolium bromide), dimethyl sulfoxide (DMSO), ethanol, Trypan Blue, sodium bicarbonate, non-essential amino acid, fatty acid reference material (F.A.M.E Mix C8–C24), carrageenan, dipyrone, and dexamethasone were purchased from Sigma-Aldrich Chemical Co. (St. Louis, MO, USA). Diazepam was purchased from Roche (Basel, Switzerland). The J774.16 cell line was acquired from ATCC. Dulbecco’s Modified Eagle’s Medium (DMEM), fetal bovine serum (FBS), penicillin, and streptomycin were all purchased from Thermo (Gibco, New York, NY, USA). Propidium iodide was purchased from BD Biosciences (California, CA, USA).

### 2.2. Oil Characterization

The fatty acid profile was characterized according to the methodology described in our previous study [18]. Briefly, approximately 15 mg (±0.1 mg) of oil was weighed in glass tubes with caps and 1.5 mL of a 0.5 M KOH solution in methanol was added. Full esterification of fatty acids was obtained using 14% BF_3_ in methanol. Extraction of fatty acid methyl esters was performed by adding 2.5 mL of saturated NaCl solution and 1 mL of hexane, followed by vortex agitation for 1 min and centrifugation at 241× *g* for 5 min. Samples were then analyzed by gas chromatography (Shimadzu GCMS-QP2010 Plus with an AOC-5000 injection system, Japan; and a J&W Scientific DB-23 column (60 m × 0.25 mm ID × 0.25 μm), Folsom, CA, USA). Quantification was achieved with a calibration curve of certified reference material (F.A.M.E Mix C8–C24).

### 2.3. Development and Physicochemical Characterization of Pequi Oil-Based Nanoemulsion (PeNE)

Pequi oil nanoemulsion (PeNE) was developed according to the methodology described in our previous study [18]. In summary, a suspension was prepared by adding egg lecithin and pequi oil in PBS. The proportions of egg lecithin and pequi oil used were 2:1 *w*/*w*. Sonication at 40 kHz under an ice bath for 3 min was used. The developed PeNE formulations were stored at 4 °C and under dark conditions until further analysis. A blank formulation (without the oil) was prepared similarly as described above.

For in vivo studies, it was necessary to prepare a more concentrated PeNE due to the limitation of volumes that could be administered (p.o.) in the groups. Therefore, the final concentration of oil and surfactant was increased proportionally 29× and 7.25× times for the doses of 100 and 400 mg/kg, respectively.

The nanodroplet hydrodynamic diameter, polydispersity index (PdI), and zeta potential were measured using ZetaSizer^®^ Nano ZS90 (Malvern, UK). All analyses were performed at room temperature, and PeNE and blanks used in in vivo assays were diluted 2.5× for these analyses.

### 2.4. PeNE Morphological Analysis

The morphological analysis of PeNE was performed by scanning electron microscopy (SEM) and transmission electron microscopy (TEM) techniques, using JSM-7001F and JEM-1011 (Jeol, Tokyo, Japan) microscopes, respectively.

Scanning electron microscopy (SEM): The samples were diluted 1:300 (*v*/*v*) in distilled water, and a 60 μL aliquot was deposited on a smooth carbon tape adhered to a stub surface, contrasted with 2% OsO_4_ vapor for 30 min, and left 24 h to dry at room temperature. After complete droplet drying, the samples were metalized with gold on a Sputter Coater (Leica, EM SCD 500, Austria), and the images were collected at a magnification of 12,000× with 15.0 kV.

Transmission electron microscopy (TEM): The samples were diluted 1:300 (*v*/*v*) in distilled water, and a 3 μL aliquot was deposited on a 200 mesh copper grid containing a Formvar ultrafilm. The samples were left for 24 h to dry at room temperature and then contrasted with 2% OsO_4_ vapor for 20 min. The images were collected at a magnification of 12,000× with 80.0 kV.

### 2.5. Cell Culture

Murine macrophages (J774.16) were cultured in Dulbecco’s Modified Eagle’s Medium (DMEM) supplemented with 10% fetal bovine serum (*v*/*v*), 1% non-essential amino acid, and 1% antibiotic at 37 °C and 5% CO_2_ a sterile Petri dishes. Cell passages and medium exchange were done every 3 days.

### 2.6. Cell Viability Assay

For cell viability assay, cells were seeded onto a 96-well culture plate at a density of 2 × 10^4^ cells per well in DMEM culture medium overnight at 37 °C, 5% CO_2_ in a humid atmosphere. Then, the medium was changed and various concentrations of PeNE, blank formulations, and free pequi oil were added (90, 180, and 360 μg/mL). The free pequi oil was diluted in ethanol previous to the treatment. The final ethanol concentration was lower than 1% per well. The plates were incubated for 24 h at 37 °C, 5% CO_2_ in a humid atmosphere.

Cell viability assay was performed using MTT (3-[4, 5-dimethylthiazol-2-yl]-2,5-diphenyltetrazolium bromide) assay. After 24 h of incubation, the treatments were removed and 150 μL of the MTT solution (0.5 mg/mL in DMEM) was added to each well. The plates were incubated for 2 h at 37 °C and 5% CO_2_ in a humid atmosphere. The culture medium was discarded and 150 μL of dimethyl sulfoxide (DMSO) was added to each well. The absorbance was monitored using a spectrophotometer with a microplate reader at 595 nm (Molecular Devices, San Jose, CA, USA). The control group was considered as 100% cell viability.

### 2.7. DNA Fragmentation Assay

The J774.16 cells exposed to PeNE and the blank at 360 µg/mL, and free oil at 180 µg/mL for 24 h, were resuspended into 1 mL of cold ethanol (70%) and stored at −20 °C for 24 h. After incubation time, the cells were washed twice with PBS 1X and 100 μL of RNase (50 μg/mL) was added for 30 min at 37 °C, protected from light. Then, 100 μL of propidium iodide (PI—20 μg/mL) in PBS was incubated for 30 min at room temperature, protected from light. The DNA fragmentation was analyzed by a flow cytometer (BD FACSVerse™, Franklin Lakes, NJ, USA) using the PI channel. A total of 10,000 events were collected per sample.

### 2.8. Association of PeNE with Macrophage Cells

For association assay, J774.16 cells were seeded at a concentration of 4 × 10^4^ cells/well in 24-well culture plates. After 24 h, cells were treated with PeNE associated with aluminum chloride phthalocyanine (AlClPt–0.02 mg/mL) at 360 µg/mL and saline solution (control). Cells were analyzed by flow cytometry (FACSVerse, BD, Franklin Lakes, NJ, USA) 30–60 and 120 min of treatment. The fluorescence of AlClPt was detected at the wavelength of 630 nm. For each sample, 10,000 events were analyzed using FlowJo^®^ vX 0.7 software.

### 2.9. In Vivo Assays

#### 2.9.1. Animals and Experimental Design

Male Wistar rats (*Rattus novergicus*) (110–250 g) were housed in temperature-controlled rooms (22–25 °C) with an alternating 12 h light–dark cycle. Water and food were available ad libitum. All experiments were conducted in accordance with the International Association for Study of Pain Guidelines, the Brazilian Guidelines for the Care of Laboratory Animals and approved by the Ethics Committee of the Institute of Biological Sciences at the University of Brasilia/Brazil (CEUA, reference number 23106.058193/2021-48). The work complied with the ARRIVE guidelines, and all efforts were made to reduce the number of animals used.

**Figure 1:**
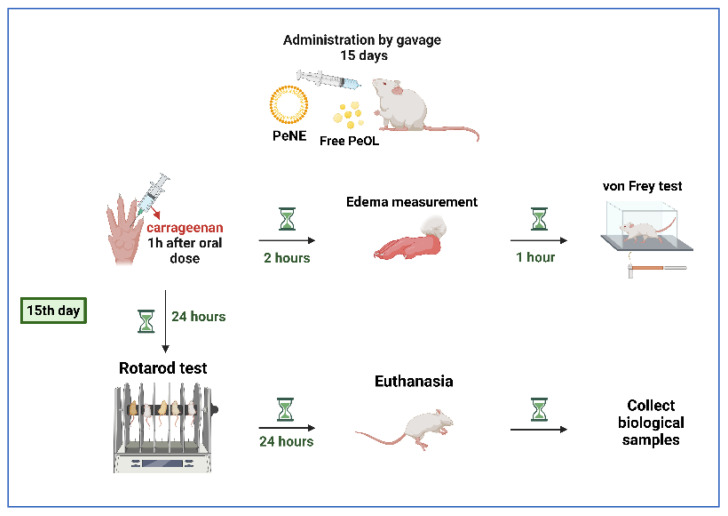
Experimental design of inflammation, hypernociception, and motor coordination assays performed with rats daily treated with PeNE (pequi oil nanoemulsion) or free pequi oil (p.o.) for 15 days.

The animals received oral administration (p.o.) of PeNE or free pequi oil (100 or 400 mg/kg) by gavage daily for 15 days (Figure 1). PeNE was diluted in PBS. Free pequi oil was dispersed in PBS and vortexed immediately before administration. “Blank groups” received volumes of blank formulations equivalent to PeNE treatment. Control groups were treated with the same regimen, but with PBS only. Four to seven animals per group were used in this experimental protocol.

On the last day, 1 h after the oral administration of PeNE, free pequi oil, and blank formulations, a carrageenan-induced acute paw inflammation model was induced in order to evaluate their potential anti-inflammatory and antinociceptive activities as described in Section 2.9.2. Later, the animals were evaluated for motor coordination (Section 2.9.3) and were euthanized for the evaluation of the treatment’s biocompatibility (Section 2.9.4) (Figure 1).

#### 2.9.2. Evaluation of Carrageenan-Induced Hypernociception and Edema in Rats

To test the anti-inflammatory and/or analgesic activity of PeNE and free pequi oil in vivo, a carrageenan-induced acute paw inflammation model was used [19,20,21]. Carrageenan (100 µg) was administered locally (hind right paw, intraplantar—i.pl.) in a volume of 100 µL per paw in all animals. One of the control groups received dexamethasone (2 mg/kg/i.p.), a potent anti-inflammatory drug, and was named as a positive control for carrageenan-induced edema evaluation. The other control group received dipyrone (120 mg/kg/i.p.), a known analgesic drug, and was named as a positive control for antinociceptive evaluation [21]. Carrageenan, dipyrone, and dexamethasone were diluted in saline, immediately before use.

The mechanical nociceptive sensitization (hypernociception) was measured with an electronic pressure meter, which consists of a handheld force transducer adapted with a 0.7-mm^2^ polypropylene tip (electronic Von Frey aesthesiometer; Insight—Brazil) [21]. In the adaptation period, rats were placed in acrylic cages (12 × 20 × 17 cm) on a wire grid floor, 15 to 20 min before beginning tests. During the test, the investigator applied the polypropylene tip perpendicularly between the five distal footpads, with a gradual increase in pressure. A tilted mirror below the grid provided a clear view of the animal’s hind paw. The test consisted of poking the hind paw to provoke a flexion reflex followed by a clear flinch response after the paw’s withdrawal. The electronic pressure meter automatically records the stimulus intensity when the paw was withdrawn (a reaction interpreted as a response to pain). The animals were tested before and after treatments. Results are expressed as intensity of hypernociception (delta force, grams), calculated by subtracting the value of the paws’ basal responses (pretreatment—zero hour) from that of carrageenan-treated paws (posttreatment—three hours after).

The edema effect of carrageenan was evaluated 2 h after its administration. The intensity of the carrageenan-induced edema was assessed by measurements of rat paw volume using a plethysmometer (UgoBasile, Gemonio, Italy) [21]. Thus, data can be obtained to help differentiate between antinociceptive and/or anti-inflammatory effects. The animals were tested after treatments, and the results are expressed as the delta microliters (µL), calculated by subtracting the value of the measurements of the treated paw (right paw) from that of the control naïve paw (left paw). Before starting the experiments, it was confirmed that there were no differences in the basal conditions of the right and left paws.

#### 2.9.3. Evaluation of Motor Coordination

Motor coordination was evaluated on a rotarod treadmill test apparatus (Insight, Brazil), which consists of a rotating rod (75 mm diameter and 40 cm height), divided into four compartments. Rats were trained to remain on the rotarod for 2 consecutive days. One day after the evaluation of PeNE and free pequi oil treatment effects on behavioral tests, individual rats were placed in the rotating rod at 20 rpm, the cutoff time was 200 s [22]. One of the control groups received diazepam (30 mg/kg/p.o.) 20 min before the assay and was named as a positive control for motor coordination evaluation [21].

#### 2.9.4. Biocompatibility Evaluation

Biocompatibility evaluations of PeNE and free pequi oil treatments (p.o.) were performed through different analyses described as follows. Clinical observations (loss of weight, diarrhea, alopecia, inappetence, motor disorders, and salivary gland secretions) were registered daily.

Biochemical and hematological analyses: Blood samples collected by cardiac puncture were used for biochemical and hematological analyses. Approximately 50 µL blood was transferred to a microtube with EDTA anticoagulant for automated complete blood count analysis in the Sysmex pocH-100iV DiffTM device. Serum levels of albumin, alanine aminotransferase (ALT), aspartate aminotransferase (AST), creatinine k, urea, glucose, lactate dehydrogenase (LDH), cholesterol, and triglycerides were measured in the chemical analyzer ChemWell-T^®^ (Labtest, Lagoa Santa, Brazil), using commercial kits (Labtest^®^) according to the manufacturer’s instructions. To obtain the serum, approximately 700 µL of whole blood was transferred to a microtube until complete coagulation and then centrifuged at 1000× *g* for 10 min at 4 °C.

Detection of ROS and NO in blood samples: Blood samples were collected by cardiac puncture using a 3-mL heparinized syringe and a 24G needle. For ROS (reactive oxygen species) detection, the blood was immediately treated with the working solution containing 400 μM 1-hydroxy-3 methoxycarbonyl-2,2,5,5-tetramethylpyrrolidine (CMH), 25 μM deferoxamine methanesulfonate salt (DF), 5 μM sodium diethyldithiocarbamate trihydrate (DETC) and heparin sodium (100 IU/mL) in 1:1 proportion. The tube was incubated at 37 °C for 30 min. After that, 50 μL of the solution was placed between two ice blocks (200 μL each) in a 1 mL decapped syringe and snapped frozen in liquid nitrogen. For the estimation of the production of NO, the concentration of nitrosyl hemoglobin (HbNO) in the blood was measured. The blood (1 mL) was transferred to 1 mL syringes, centrifuged at 2000 rpm for 5 min, and frozen in liquid nitrogen. All samples were stored at −80 °C until the electron paramagnetic resonance (EPR) measurements were performed.

EPR measurements were performed in an EPR spectrometer EMX plus (Bruker, Germany), by using X-band (9 GHz) and a high-resolution cavity (ER 4119HS, Bruker, Germany). For ROS and NO detection, the samples were transferred under liquid nitrogen to a finger dewar (Noxygen, Germany), and the spectra were recorded at 77K. For ROS detection, the instrumental settings were 2 mW microwave power, 5G modulation amplitude, 100 kHz of modulation frequency, and 200G sweep width. To evaluate the ROS production, we used the first derivative spectrum peak-to-peak amplitude substituted in a calibration curve made by using the nitroxide radical CP· diluted in Krebs HEPES Buffer (KHB) at a dose range of 0, 5, 10, 50, 100, and 200 μM. In this concentration range, a linear calibration curve was obtained, and all the recorded data were within this calibration range.

The amount of detected NO was determined from the calibration curve for the intensity of the EPR signal of erythrocytes treated with known concentrations of nitrite (1–25 μM) and Na_2_S_2_O_4_ (20 mM). EPR spectrometer settings were as follows: 10 mW microwave power, 5G modulation amplitude, 100 kHz modulation frequency, and 320 G sweep width.

Histopathological analysis: The spleen, kidney, liver, lung, pancreas, and heart were collected for routine histopathological examination. The samples were fixed with 10% buffered formalin, passed through the ethanol-xylene series for dehydration and diaphanization, and then embedded in liquid paraffin. Sections of 5 μm thickness were obtained in a manual microtome and stained with hematoxylin-eosin (H&E) for light microscope analysis.

### 2.10. Statistical Analysis

Statistical differences between control and treated groups were evaluated by the analysis of variance (ANOVA) and Tukey post hoc test at a significance level of 0.05 using Graph Pad Prism 8.0 (GraphPad Software, La Jolla, CA, USA) after verifying the normality of data with Shapiro–Wilk. All values were expressed as mean ± standard error of the mean (SEM) and a value of *p* < 0.05 was considered statistically significant.

## 3. Results

### 3.1. Pequi Oil Characterization

Analysis of pequi oil fatty acid profile showed the presence of unsaturated fatty acids and saturated fatty acids, mainly oleic acid (47.91%) and palmitic acid (35.22%). Lower content of palmitate esters (7.89%) and oleate esters (8.99%) was also found.

### 3.2. Characterization of Pequi Oil-Based Nanoemulsion (PeNE)

The physicochemical parameters of the obtained PeNE were evaluated (Table 1). The PeNE used for the in vitro studies presented an average hydrodynamic diameter of 124.2 ± 2.4 nm, polydispersity index (PdI) of 0.244 ± 0.025, and negative zeta potential of −15.0 ± 1.7 mV. The blank formulation (without the oil) showed hydrodynamic diameter and PDI similar to the PeNE, but with a less negative value for the zeta potential (−1.68 mV). The more concentrated PeNE formulations used for in vivo studies presented an average hydrodynamic diameter in the range of 230 to 260 nm, PdI of 0.234 to 0.239, and negative zeta potential in the range of −11.93 to 14.6 mV (Table 1).

**Figure 2:**
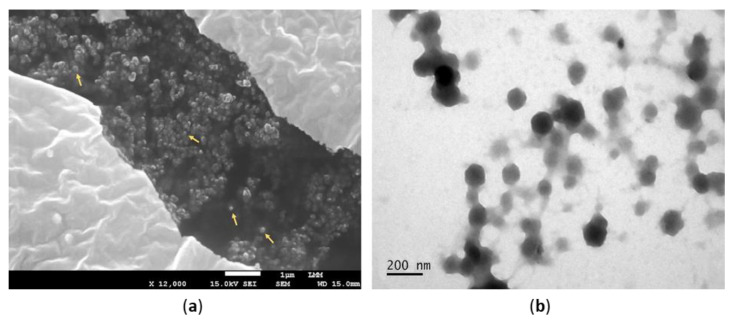
Representative ultramicrographs of pequi oil-based nanoemulsions (PeNE), acquired via (**a**) scanning and (**b**) transmission electron microscopy. Yellow arrows indicate PeNE nanodroplets in panel (**a**).

The PeNE ultrastructural analyses were obtained by scanning (SEM) and transmission (TEM) electron microscopy, allowing complementary data on the morphological properties of the nanosystem. As opposed to the Photon Correlation Spectroscopy (PCS) technique, which considers the hydrated particle size (hydrodynamic diameter), SEM and TEM provide its dry size, given the sample processing. The representative ultramicrographs (Figure 2), acquired by both techniques, indicated spherical or quasi-spherical morphologies for PeNE nanodroplets.

### 3.3. Association, Cytotoxicity, and Genotoxicity of PeNE and Free Pequi Oil on Macrophage Cells (J774.16)

Macrophages are known to be cells that play key roles in inflammatory and anti-inflammatory processes [23]. Therefore, before proceeding to in vivo studies, the association, cytotoxicity, and genotoxicity of PeNE and free pequi oil on macrophages (J774.16) were analyzed in vitro.

**Figure 3:**
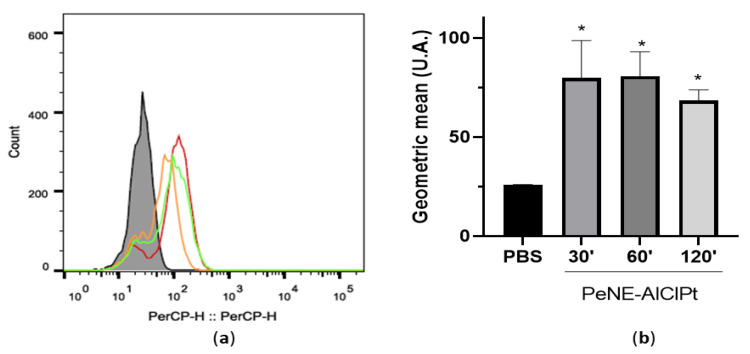
Association of nanoemulsions in macrophages measured by flow cytometry (10,000 events/sample) in J774-1 cells exposed to NePE-AlClPt for 30–60 and 120 min at 360 µg/mL. PBS was used as the control. (**a**) Histogram representation. Legend: not-treated cells (gray line); NePE-AlClPt treated cells for 30 min (green line), 60 min (red line), and 120 min (orange line). (**b**) Graphical representation. One-way ANOVA: significant difference between groups vs. control * *p* < 0.05 (Tukey post hoc test).

Figure 3 shows the association of PeNE with J774.16 cells after 24 h of incubation. It is interesting to note that NePE interacts with macrophages in the first 30 min of exposure. No time-dependent association of PeNE with J774.16 cells was observed (Figure 3). After 120 min, the fluorescence tends to decrease when compared to early times (30 and 60 min), suggesting a reduction of the association, but no significant difference was detected.

**Figure 4:**
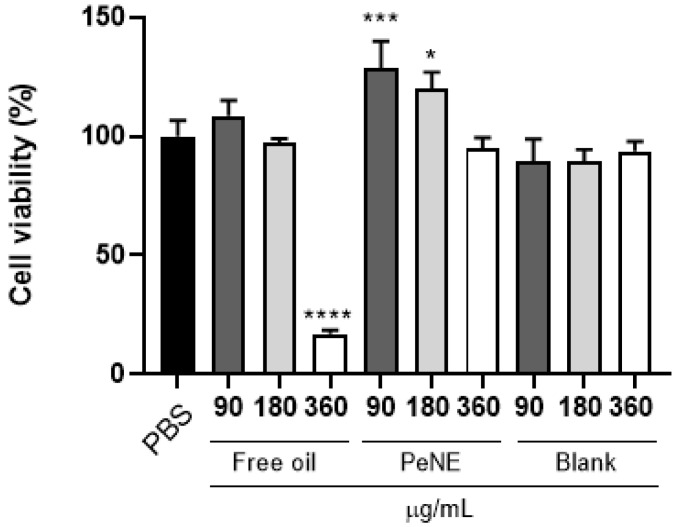
Cytotoxicity of pequi oil-based nanoemulsion (PeNE), free pequi oil, and the blank (egg lecithin without oil) formulation at 90, 180 and 360 µg/mL on the viability of macrophage cells (J774.16) after exposure for 24 h. The control group was treated with PBS. The assay was carried out using the MTT method. The values are expressed as mean ± SEM. One-way ANOVA: significant difference compared to the control group * *p* < 0.05, *** *p* < 0.001, and **** *p* < 0.0001.

The next step involved the evaluation of PeNE and free pequi oil cytotoxicity on J774.16 cells. A slightly significant increase in cell viability (28 and 20%, respectively; *p* < 0.05) was observed in cells treated with 90 and 180 µg/mL of PeNE when evaluated with the MTT assay. In addition, a significant reduction of 74% in cell viability was observed in cells treated with free pequi oil only at the higher concentration evaluated (360 µg/mL) (Figure 4).

**Figure 5:**
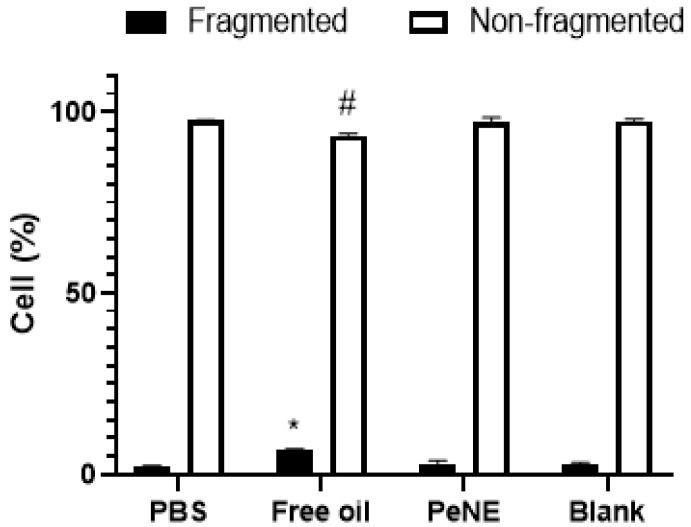
DNA fragmentation measured by flow cytometry (10,000 events/sample) in macrophage cells (J774.16) exposed to NePE (360 µg/mL), blank, and free pequi oil (180 ug/mL) for 24 h. Phosphate buffer and ethanol were used as controls. Two-way ANOVA: significant difference among fragmented groups * (*p* < 0.05) and non-fragmented group ^#^ (*p* < 0.05) (Tukey post hoc test).

The concentrations of 180 and 360 µg/mL were chosen to evaluate the genotoxicity of free pequi oil and PeNE, respectively, on J774.16 cells through the analysis of the DNA fragmentation pattern of treated cells. Figure 5 shows that PeNE did not induce significant DNA fragmentation after 24 h of exposure. Free pequi oil induced a slight DNA fragmentation of 6.5% (*p* < 0.05) when compared to the control group.

### 3.4. Evaluation of Anti-Inflammatory and Analgesic Effects of PeNE and Free Pequi Oil In Vivo

To evaluate the anti-inflammatory and antinociceptive effects of PeNE and free pequi oil, daily oral administrations with different concentrations of PeNE and free pequi oil (100 and 400 mg/kg) were carried out for 15 days, before the induction of a local (paw) acute inflammation with carrageenan.

**Figure 6:**
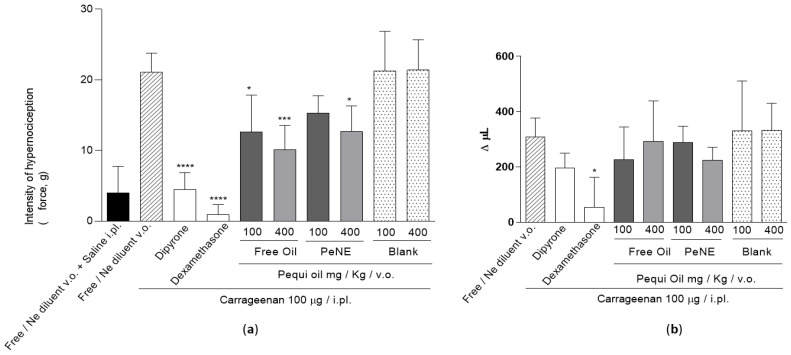
Carrageenan-induced hypernociception in rats is inhibited by the free or nanoencapsulated pequi oil. The figure shows the hypernociception (**a**) or edema (**b**) induced by a single i.pl. injection of carrageenan (100 g/paw) and the effect of pre-treatment with free/Ne diluent or dipyrone (120 mg/kg/i.p.) or dexamethasone (2 mg/kg/i.p.) or free oil (100 and 400 mg/kg/p.o.) or nanoencapsulated pequi oil (PeNE 100 and 400 mg/kg/p.o.) or the blank. PeNE, free pequi oil, and the blank were administered daily for 15 days prior to carrageenan injection. The data are expressed as mean ± SEM; (*n* = 4–7). One-way ANOVA with Tukey’s comparison: significant difference compared to the oil/Ne diluent plus carrageenan group *** *p* < 0.001, **** *p* < 0.0001 (**a**) and * *p* < 0.05 (**b**).

Figure 6 shows the oral treatment effects of PeNE or free pequi oil on behavioral tests. PeNE (100 or 400 mg/kg, both) reduced the hypernociception by 27 and 40%, respectively (*p* < 0.05); while free pequi oil reduced it by 40 and 52%, respectively (*p* < 0.05) (Figure 6a). The control treatments with dipyrone, a known analgesic drug, and dexamethasone, a potent anti-inflammatory drug, showed a reduction in hypernociception by 79 and 96%, respectively (Figure 6a).

**Figure 7:**
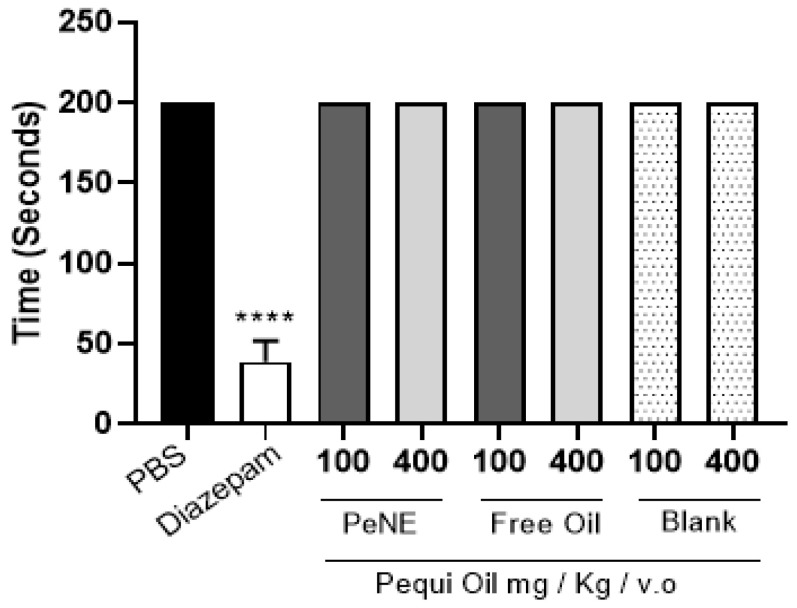
Rota-rod test in rats pre-treatment with free oil (100 and 400 mg/kg/p.o.), nanoencapsulated pequi oil (PeNE 100 and 400 mg/kg/p.o.), blank, or diazepam. The control group was treated with PBS. PeNE, free pequi oil, and the blank were administered daily for 15 days prior to carrageenan injection. Latency to falling (in seconds) on the rotating rod. The data are expressed as mean ± SEM; (*n* = 4–7). **** *p* < 0.0001 compared to control group; one-way ANOVA.

Besides showing an antinociceptive effect, the treatment with PeNE or free pequi oil (same doses) did not show an effect against the carrageenan-induced edema (Figure 6b). As expected, dipyrone did not show an anti-inflammatory effect and dexamethasone inhibited the edema by 82% (*p* < 0.05). The antinociceptive effect observed by oral treatments with PeNE or free pequi oil is not due to motor coordination impairment since no significant difference was observed among treatments (Figure 7).

### 3.5. Biocompatibility Analysis of PeNE and Free Oil Oral Treatment in In Vivo Model

**Figure 8:**
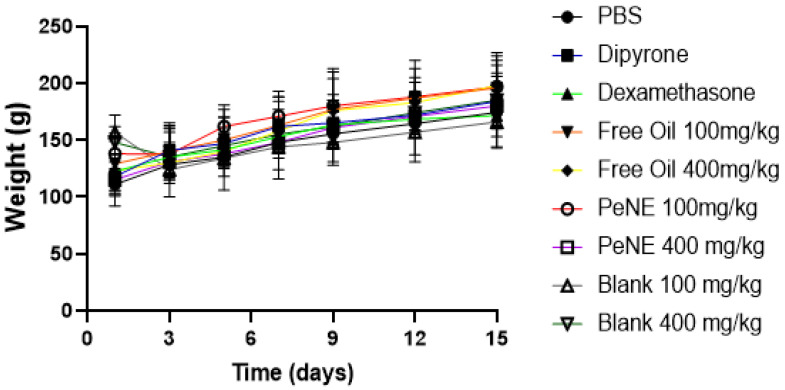
Weight of the animals during the 15 days of treatment with free oil (100 and 400 mg/kg/p.o.), nanoencapsulated pequi oil (PeNE 100 and 400 mg/kg/p.o.), blank, dipyrone (120 mg/kg/i.p.), dexamethasone (2 mg/kg/i.p.) and PBS, the negative control group.

The oral administration of PeNE and free pequi oil samples did not induce clinical or behavioral alterations such as inappetence, motor dysfunction, salivary gland secretions, and hair loss throughout the entire experimental period in all animals investigated. Further, PeNE and free pequi oil samples did not induce significant modification in the animal weight in the treated groups when compared to their corresponding controls (Figure 8).

The biochemical (Table 2), cholesterol and triglycerides (Table 3), erythrogram (Table 4), and leukogram (Table 5) of treated animals showed no significant alterations when compared to their corresponding controls. The presence of ROS and NO in blood samples of all treated animals was analyzed, showing no significant difference when compared to the control group (data not shown).

**Figure 9:**
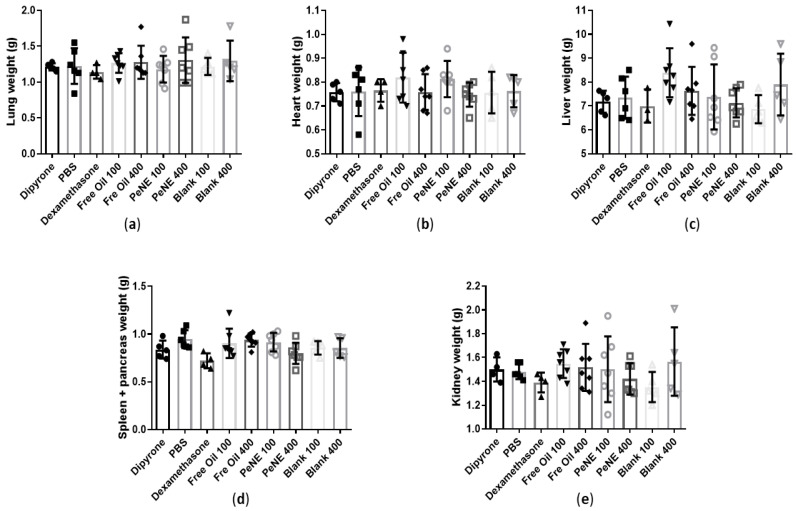
Evaluation of the weight of the organs of animals pre-treated with free oil (100 and 400 mg/kg/p.o.) or nanoencapsulated pequi oil (PeNE 100 and 400 mg/kg/p.o.) or the blank or control groups for 15 days. (**a**) Lung weight; (**b**) heart weight; (**c**) liver weight; (**d**) spleen and pancreas weight; (**e**) kidney weight. Statistics: ordinary one-way ANOVA. All data are presented as mean ± SD (*n* = 4–7).

The macroscopic aspects of evaluated organs (liver, lungs, spleen, pancreas, heart, and kidneys) were preserved and similar to the control group (Figure S1, Supplementary Materials). Regarding the organ’s weight, no significant difference was observed among the experimental groups when compared to the control (Figure 9).

**Figure 10:**
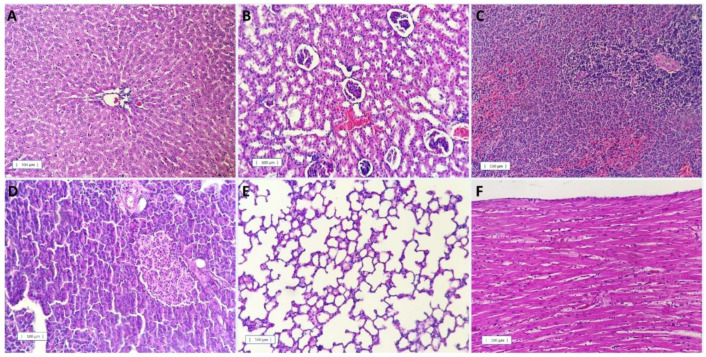
Histopathological evaluation. Representative photomicrographs of (**A**) liver, (**B**) kidney, (**C**) spleen, (**D**) pancreas, (**E**) lung, and (**F**) hearts of animals pre-treated with free oil (100 and 400 mg/kg/p.o.), nanoencapsulated pequi oil (PeNE 100 and 400 mg/kg/p.o.), or the blank for 15 days. The organs of all groups showed standard morphology. Scale bar 100 µm.

After a careful histopathological examination of the selected organs of all treated animals, no obvious changes were identified in the parenchyma, with all groups showing similar aspects when compared to the control group. Figure 10 illustrates a representative histological aspect of each organ evaluated.

## 4. Discussion

The present study aimed to investigate the biocompatibility, anti-inflammatory, and antinociceptive activities of PeNE and free pequi oil in in vitro and in vivo models. Before the beginning of the investigations, the lipid profile of pequi oil used was analyzed. It is known that the lipid profile of pequi oil can vary according to environmental factors (e.g., temperature, region, soil, climate) [14,25,26]. In the present study, the fatty acid profile found for pequi oil was similar to the profiles reported in the literature [14,25,26], showing the main presence of oleic and palmitic acids.

The next step consisted of the preparation and characterization of pequi oil nanoemulsions (PeNE). Nanodroplet characterization is considered a relevant prior step to ensure their suitability for in vitro and in vivo studies [27]. The obtained PeNE showed spherical or quasi-spherical morphologies with hydrodynamic diameters <200 nm, which is in the range expected for phosphatidylcholine-based nanoemulsions (150–300 nm) [28]. In addition, it was possible to obtain a nanoemulsion suspension with monodisperse nanodroplets (PDI < 0.260) [27] and negative zeta potential. All these characteristics are in accordance with previously published studies [18,29]. Considering that, for in vivo models, the dose is calculated based on body weight and that there is a limited volume that can be used per administration in the oral route [30], it was necessary to increase the quantity of pequi oil and surfactant (proportionally) for the formulations used in in vivo studies. It is known that increases in the total concentration of nanoemulsion oil phase components can result in nanodroplets with different physicochemical characteristics [11,29]. Herein, it was observed that concentrated PeNE formulations maintained monodisperse PDI and negative zeta potential characteristics. In addition, besides the increase in the hydrodynamic diameter, concentrated PeNE formulations still showed values <260 nm.

Through the simulation of biological conditions, in vitro analyses generate results that aid in the prediction of possible effects of bioactive compounds in in vivo models, enabling a reduction in laboratory financial expenses and work time [31]. In vitro biocompatibility tests are essential to expand knowledge about the cytotoxicity profile and determine the feasibility of using nanostructures in vivo [32]. In addition to cytotoxicity assays, investigations on the potential genotoxicity of nanostructured compounds are relevant, since alterations in genetic material can lead to mutagenicity, unregulated cell growth (carcinogenicity), or even induce cell death [33]. Therefore, before starting the in vivo assays, the association, cytotoxicity, and genotoxicity of PeNE to macrophage cells (J774.16) were evaluated in vitro. Macrophages are cells that have a stimulus-dependent polarization capacity, which allows these cells to play dual roles in the orchestration of inflammation, through the expression of pro-inflammatory (M1) and anti-inflammatory (M2) phenotypes [23]. Present data revealed that PeNE nanodroplets were able to be effectively associated with macrophage cells without inducing cytotoxic or genotoxic outcomes (Figure 3, Figure 4 and Figure 5).

The inflammatory process is characterized as a mechanism of physiological action, aiming for the defense of the organism by producing several inflammatory mediators that will interact in numerous cells of the body in order to eliminate the agent causing inflammation and to start the process of local tissue recovery [34]. When the acute inflammatory response is persistent, the body is induced to produce inflammatory mediators constantly. This process can destroy healthy tissues and cause several types of damage to the body, which can result in disorders of inflammatory origin such as cardiovascular diseases, some types of cancers, intestinal diseases, diabetes, obesity, and others [34].

Analgesics and anti-inflammatory drugs are important tools to aid in the treatment of persistent inflammatory processes. Nevertheless, long-term use of such drugs can lead to undesired side effects [35]. Plants are relevant sources of bioactive molecules able to regulate inflammatory processes [3]. Thus, the search for analgesic and/or anti-inflammatory drugs developed from natural oils associated with nanostructures, which improve their bioavailability and enhance their use in alternative and/or complementary anti-inflammatory and antinociceptive treatments, is important.

The carrageenan-induced edema in vivo assay is traditionally used for screening and development of new compounds, and it is one of the most popular tests used due to its high sensitivity and reproducibility for anti-inflammatory and analgesic drugs [20,36]. Fixed natural oils, such as pequi oil, have demonstrated potential anti-inflammatory, anti-edema, and antinociceptive activities [5,6,14,15,16,17]. Junior and co-workers [5] have described significant anti-inflammatory and antinociceptive effects of free pequi oil with a single oral administration with doses of 700 mg/kg and 1000 mg/kg respectively. In the present study, daily PeNE or free pequi oil oral treatments for 15 days did not induce significant anti-edema and anti-inflammatory effects (Figure 6). Nevertheless, significant antinociceptive effects were observed in PeNE and free pequi oil treatments with lower concentrations (100 and 400 mg/kg) than described by Junior and co-workers [5] (Figure 6). Treatment regimen and dose have an important impact on anti-inflammatory and antinociceptive outcomes. Besides administering PeNE and free pequi oil for a longer period (15 days) herein, the dose used was not enough to achieve the anti-inflammatory effect expected. Further studies are needed to evaluate if oral administration of higher doses (>700 mg/kg) of PeNE would show anti-inflammatory effects. Regarding antinociceptive effects, present data suggest that administration of PeNE and free pequi oil for longer periods (15 days) could induce analgesic effects with lower doses (100–400 mg/kg) than described in the literature for a single dose treatment (700–1000 mg/kg) [5]. It is important to highlight that the analgesic effect observed herein was not due to impairment of motor coordination, since this parameter was similar to non-treated groups (Figure 7). To the best of our knowledge, the present work is the first to show the antinociceptive effect of pequi oil carried by a nanoemulsion system.

The use of nanostructured drug delivery systems for hydrophobic bioactive compounds is an interesting approach to overcoming administrative limitations and improving their biodistribution, stability, and efficiency, along with the reduction of possible side effects [6]. Coutinho and co-workers [6] showed a significant anti-inflammatory effect of a pequi oil nanoemulsion in a pleural inflammation model, but no significant effect was observed with the oil administered in a free form, therefore emphasizing that the use of nanostructures has a decisive impact on the physiologic effect of pequi oil. In the present work, the administration of pequi oil in a nanoemulsion system showed similar anti-inflammatory and antinociceptive effects when compared to free pequi oil. Nevertheless, it is worth mentioning that the use of nanoemulsion did not interfere with the antinociceptive effect of pequi oil, suggesting that such an approach could still be valuable for the conservation of bioactive compounds in preventive/therapeutic formulations [8,11].

During pre-clinical screenings of new nanostructures aimed at biomedical/nutraceutical applications, biocompatibility tests using in vivo models are fundamental preliminary assessments performed in order to better understand the toxicity effects of nanomaterials in more complex biological systems [32]. Since pequi oil has already been used in traditional medicine for decades [5,6], there is a chance that its regular use could trigger no or mild adverse effects. Biocompatibility evaluations of free pequi oil oral administration have already been described for in vivo models and also clinical studies, showing no significant or dose-dependent mild alterations [16,17,37]. Present data corroborate the findings in previously published studies. In the present work, PeNE or free pequi oil treatments were seen to be biocompatible, showing no significant alterations in body weight, motor coordination, blood cell counts, biochemical parameters, blood levels of ROS and NO, organ anatomy and weight, and in histopathology evaluations (Figure 8, Figure 9 and Figure 10 and Table 2, Table 3, Table 4 and Table 5). Interestingly, the use of pequi oil in a nanoemulsion system did not induce significant alterations in the evaluated parameters, suggesting that this delivery system is safe to be administered in the doses and regimens evaluated herein.

## 5. Conclusions

The results of this study revealed that PeNE associates with macrophages, maintaining their cell viability, and with no induction of DNA fragmentation in in vitro assays. Moreover, daily oral PeNE or free pequi oil administrations for 15 days induced a significant reduction in carrageenan-induced paw mechanical hypernociception, independently of an anti-inflammatory effect pattern evaluated. In addition, to the best of our knowledge, the present work is the first to show the antinociceptive effect of pequi oil carried by a nanoemulsion system. PeNE or free pequi oil treatments were observed to be biocompatible, showing no significant alterations in body weight, motor coordination, blood cell counts, biochemical parameters, blood levels of ROS and NO, organ anatomy and weight, and in histopathology evaluations. These promising results highlight the potential use of PeNE and free pequi oil as a biocompatible complementary preventive/therapeutic approach to be employed, along with conventional treatments, for analgesic treatments in the future.

## 6. Patents

G. A. Joanitti and R. B. Azevedo are the named inventors of the following patent application (patent: BR 10 2017 025294 9, 24 November 2017, Brazilian Patent Office [Instituto Nacional da Propriedade Industrial—INPI]) regarding the development and use of pequi oil-based nanoemulsions. This patent does not represent a direct conflict of interest to the reported data in this manuscript.

## Figures and Tables

**Table 1:** Physicochemical characteristics of the PeNE formulations. The results are reported as the average of three independent measurements (*n* = 3 ± SD).

Sample	Hydrodynamic Diameter (nm)	PDI	Zeta Potential (mV)
PeNE	124.20 ± 2.40	0.24 ± 0.030	−15.00 ± 1.70
Blank	157.90 ± 12.12	0.24 ± 0.010	−1.68 ± 0.08
PeNE concentrated A	231.10 ± 3.07	0.239 ± 0.021	−11.93 ± 1.28
PeNE concentrated B	259.00 ± 1.57	0.234 ± 0.014	−14.6 ± 2.85
Blank concentrated	249.36 ± 17.81	0.467 ± 0.214	−3.88 ± 1.23

Legend: PeNE and blank formulations were used in in vitro assays. PeNE concentrated A used in in vivo tests at a concentration of 100 mg/kg, PeNE concentrated B, and blank concentrate at a concentration of 400 mg/kg. All formulations had pH = 7.

**Table 2:** Biochemical analysis from the blood samples of rats after oral treatment with PeNE or free pequi oil for 15 days.

Sample	Albumin (g/dL)	ALT (U/L)	AST (U/L)	Creatinine (mg/dL)	Glucose (mg/dL)	Urea (mg/dL)
PBS	2.59 ± 0.20	54.33 ± 3.78	200.7 ± 60.75	0.54 ± 0.14	179.00 ± 18.33	43.25 ± 4.34
Dexamethasone	2.95 ± 0.16	49.00 ± 11.60	123.80 ± 33.35	0.65 ± 0.08	333.7 ± 66.12 *	41.50 ± 11.45
Dipyrone	2.56 ± 0.24	47.00 ± 8.60	121.2 ± 28.80	0.53 ± 0.03	204.40 ± 84.83	40.60 ± 4.33
PeNE 100 mg/kg	2.85 ± 0.27	48.00 ± 10.41	200.5 ± 116.50	0.64 ± 0.10	288.30 ± 131.80	41.71 ± 4.82
PeNE 400 mg/kg	2.88 ± 0.12	54.43 ± 8.28	159.70 ± 52.73	0.61 ± 0.07	280.70 ± 101.5	40.43 ± 3.82
Oil free 100 mg/kg	2.75 ± 0.39	57.67 ± 13.09	176.20 ± 88.27	0.60 ± 0.12	254.70 ± 126.40	45.86 ± 7.73
Oil free 400 mg/kg	2.84 ± 0.17	59.29 ± 18.49	154.00 ± 61.26	0.58 ± 0.11	185.60 ± 21.24	41.71 ± 8.15
Blank 100 mg/kg	2.84 ± 0.04	52.80 ± 4.97	139.8 ± 36.26	0.69 ± 0.09	338.00 ± 101.4 *	49.20 ± 7.72
Blank 400 mg/kg	2.94 ± 0.16	50.80 ± 12.77	166.00 ± 70.20	0.64 ± 0.09	390.40 ± 104.3 *	44.60 ± 15.52
Reference values ^a^	3.40–4.80 ± 0.40	18.00–45.00 ± 7.00	74.00–143.00 ± 20.00	0.20–0.50 ± 0.10	70.00–208.00 ± 38.00	12.30–24.60 ± 2.90

^a^ Reference values of male Wistar rats (8–16 weeks old) [24]. Legend: ALT: alanine aminotransferase; AST: aspartate aminotransferase. Statistics: Ordinary one-way ANOVA. Asterisks indicate statistically significant differences as * *p* < 0.05. All data are presented as mean ± SD (*n* = 4–7).

**Table 3:** Cholesterol and triglycerides analyses from the blood samples of rats after oral treatment with PeNE or free pequi oil for 15 days.

Sample	Cholesterol (mg/dL)	Triglycerides (mg/dL)
PBS	58.75 ± 9.21	70.75 ± 6.39
Dexamethasone	47.75 ± 8.77	63.25 ± 29.64
Dipyrone	49.60 ± 5.41	51.20 ± 12.64
PeNE 100 mg/kg	61.57 ± 8.14	61.50 ± 15.93
PeNE 400 mg/kg	55.29 ± 6.94	60.29 ± 26.39
Oil free 100 mg/kg	57.14 ± 8.23	51.67 ± 19.58
Oil free 400 mg/kg	52.00 ± 7.02	51.71 ± 15.48
Blank 100 mg/kg	56.00 ± 5.70	67.00 ± 33.18
Blank 400 mg/kg	59.60 ± 5.32	50.40 ± 20.27
Reference values ^a^	37.00–85.00 ± 13.00	20.00–114.00 ± 21.00

^a^ Reference values of male Wistar rats (8–16 weeks old) [24].

**Table 4:** Erythrogram analyses in male Wistar rats after oral treatment with PeNE or free pequi oil for 15 days.

Sample	RBC (×10^6^/μL)	HGB (g/dL)	HCT (%)	MCV (fL)	MCH (pg)	MCHC (g/dL)	RDW-CVRL (%)
PBS	7.083 ± 1.26	14.27 ± 2.34	41.35 ± 6.66	58.57 ± 2.49	20.20 ± 0.91	34.47 ± 0.59	17.78 ± 3.35
Dexamethasone	7.64 ± 0.61	16.23 ± 1.08	46.90 ± 2.59	61,50 ± 1.63	21.25 ± 0.47	34.58 ± 0.53	17.28 ± 4.80
Dipyrone	6.99 ± 0.42	14.60 ± 1.00	43.60 ± 5.80	59.52 ± 1.95	20.88 ± 0.55	35.10 ± 1.12	18.62 ± 1.71
PeNE 100 mg/kg	6.67 ± 1.83	13.70 ± 3.86	39.60 ± 10.89	58.10 ± 3.39	20.53 ± 0.88	34.51 ± 0.65	18.80 ± 4.10
PeNE 400 mg/kg	7.50 ± 0.436	15.34 ± 1.01	44.33 ± 2.19	59.14 ± 2.07	20.46 ± 0.78	34.61 ± 0.68	18.96 ± 3.04
Oil free 100 mg/kg	7.31 ± 0.68	15.10 ± 1.45	44.33 ± 4.136	60.61 ± 0.48	20.64 ± 0.28	34.04 ± 0.54	16.99 ± 2.52
Oil free 400 mg/kg	7.32 ± 0.35	15.20 ± 0.69	44.16 ± 2.24	60.27 ± 1.74	20.76 ± 0.70	34.34 ± 0.35	16.87 ± 2.60
Blank 100 mg/kg	6.57 ± 1.22	14.04 ± 2.49	40.82 ± 6.71	62.38 ± 2.54	21.40 ± 0.55	34.34 ± 0.64	16.86 ± 2.40
Blank 400 mg/kg	7.26 ± 0.55	15.24 ± 1.18	43.66 ± 3.65	60.14 ± 1.92	21.00 ± 0.48	34.90 ± 0.41	19.64 ± 3.59
Reference values ^a^	7.27–9.65 ± 0.67	13.70–17.60 ± 1.00	39.60–52.50 ± 3.50	49.90–57.90 ± 2.4	17.10–20.40 ± 0.80	32.90–37.50 ± 1.20	11.10–15.2 ± 1.10

^a^ Reference values of male Wistar rats (8–16 weeks old) [24]. Legend: RBC—red blood cells; HGB—hemoglobin; HCT—hematocrit; MCV—mean corpuscular volume; MCH—mean corpuscular hemoglobin; MCHC—mean corpuscular hemoglobin concentration; RDW-CV—red blood cell distribution width as a coefficient of variation. Statistics: ordinary one-way ANOVA. All data are presented as mean ± SD (*n* = 4–7).

**Table 5:** Leukogram analyses in male Wistar rats after oral treatment with PeNE or free pequi oil for 15 days.

Sample	WBC (×10^3^/μl)	W-SCR (%)	W-LCR (%)	W-SCC (× 10^3^/µL)	W-LCC (×10/µL)
PBS	4.78 ± 1.26	60.47 ± 9.39	39.53 ± 9.39	2.90 ± 0.95	1.88 ± 0.58
Dexamethasone	10.95 ± 2.90	55.78 ± 7.40	44.23 ± 7.40	6.10 ± 1.89	4.85 ± 1.51
Dipyrone	3.76 ± 1.09 **	59.60 ± 9.25	40.40 ± 9.25	2.26 ± 0.76 **	1.50 ± 0.53 **
PeNE 100 mg/kg	7.95 ± 3.70	63.29 ± 4.03	36.71 ± 4.03	5.07 ± 2.55	2.88 ± 1.19
PeNE 400 mg/kg	7.07 ± 3.92	58.33 ± 5.58	41.67 ± 5.58	4.15 ± 2.45	2.86 ± 1.55
Oil free 100 mg/kg	7.07 ± 3.46	57.77 ± 3.75	41.50 ± 4.24	4.22 ± 2.23	2.950 ± 1.33
Oil free 400 mg/kg	7.52 ± 3.90	59.61 ± 6.25	40.39 ± 6.25	4.54 ± 2.47	2.98 ± 1.19
Blank 100 mg/kg	5.70 ± 3.52	64.40 ± 3.77	35.60 ± 3.77	3.70 ± 2.26	2.00 ± 1.29
Blank 400 mg/kg	5.02 ± 1.66	55.32 ± 6.29	44.68 ± 6.29	2.76 ± 0.90	2.26 ± 0.91
Reference values ^a^	1.96–8.25 ± 0.67	66.60–90.30 ± 6.30	6.20–26.70 ±5.50	-	-

^a^ Reference values of male Wistar rats (8–16 weeks old) [24]. Legend: WBC—total white blood cells; W-SCR—small cell ratio (lymphocytes); W-LCR—large cell. Ratio (neutrophils); the absolute number of small (W-SCC) and large (W-LCC) cells; statistics: ordinary one-way ANOVA. Asterisks indicate statistically significant differences as ** *p* < 0.01. All data are presented as mean ± SD (*n* = 4–7).

## Data Availability

Will be provided when requested.

## Supplementary Materials

The following supporting information can be downloaded at: https://www.mdpi.com/article/10.3390/nano12234260/s1, Figure S1: Macroscopic photos of the organs of a representative animal of each experimental group, containing lung, heart, kidneys, pancreas associated with the spleen, liver respectively.
