# Supplementary material for: Evaluation of Biocompatibility, Anti-Inflammatory, and Antinociceptive Activities of Pequi Oil-Based Nanoemulsions in In Vitro and In Vivo Models
Source: Nanomaterials (Basel). 2022 Nov 30;12(23):4260. doi: 10.3390/nano12234260 (PMC9740267; doi:10.3390/nano12234260)
Supplement: Supplementary file 1 [file nanomaterials-12-04260-s001.zip › nanomaterials-1975737-supplementary.pdf]

nanomaterials

MDPI

Supplementary Material

Evaluation of Biocompatibility, Anti-Inflammatory, and Antinociceptive Activities of Pequi Oil-Based Nanoemulsions in In Vitro and In Vivo Models

Andréia C. Pinheiro [1,2], Alicia S. Ombredane [1,2], Willie O. Pinheiro [1,3], Laise R. Andrade [1,2], Vitória R. P. Silva [1], Gisela J. Felice [1], Débora S. Alves [4], Aryanne F. Albernaz [3], Ariane P. Silveira [5], Milena C. F. Lima [6], Valdir F. Veiga-Junior [6], Thamis F. S. Gomes [1,2], Emanuel A. M. Damasceno [7], Fabiane H. Veiga-Souza [4,8], Paulo E. N. Souza [9], Sônia N. Báo [5], Eliza C. B. Duarte [10], Marcella L. B. Carneiro [1,11], Ricardo B. Azevedo [2], Mani I. Funez [3,12], Graziella A. Joanitti [1,2,3,*]

1 Laboratory of Bioactive Compounds and Nanobiotechnology (LBCNano), Campus Universitário—Centro Metropolitano, University of Brasilia, Ceilândia Sul, Brasília 72220-275, DF, Brazil; andreiacp@gmail.com (A.C.P.); alicia.ombredane@unb.br (A.S.O.); willie.oliveira@unb.br (W.O.P.); laise.andrade@unb.br (L.R.A.); 170115721@aluno.unb.br (V.R.P.S.); felicegis@gmail.com (G.J.F.); thamisunb@gmail.com (T.F.S.G.);
2 Post-Graduation Program in Nanoscience and Nanobiotechnology, Institute of Biological Sciences, Campus Universitário Darcy Ribeiro, University of Brasilia, Brasília 70910-900, DF, Brazil; razevedo@unb.br (R.B.A)
3 Postgraduate Program in Health Sciences and Technologies, School of Ceilândia, Campus Universitário—Centro Metropolitano, University of Brasilia, Ceilândia Sul, Brasília 72220-275, DF, Brazil; aryannealbernaz@gmail.com (A.F.A.); mani@unb.br (M.I.F.);
4 Laboratory of Protein Chemistry and Biochemistry, Department of Cell Biology, Institute of Biological Sciences, Campus Universitário Darcy Ribeiro, University of Brasilia, Brasília 70910-900, DF, Brazil; 200003887@aluno.unb.br (D.S.A.); fhveiga@unb.br (F.H.V-S.);
5 Laboratory of Microscopy and Microanalysis, Department of Cellular Biology, Institute of Biological Sciences, Campus Universitário Darcy Ribeiro, University of Brasilia, Brasília 70910-900, DF, Brazil; pandolfo.ariane@gmail.com (A.P.S.); snbao@unb.br (S.N.B.)
6 Chemistry Section, Military Institute of Engineering, Praça Gen. Tibúrcio, 80, Praia Vermelha, Rio de Janeiro 22290-270, RJ, Brazil; mcampelo@ufam.edu.br (M.C.F.L.); valdir.veiga@gmail.com (V.F.V-J.);
7 Nucleus of Cytopathology and Anatomic Pathology, Regional Hospital of Taguatinga, Health Department, Taguatinga, Brasilia 72120-970,DF, Brazil; damascenoemanuel@gmail.com (E.A.M.D.)
8 Pharmaceutical Sciences School, Facultyl of Ceilândia, Campus Universitário—Centro Metropolitano, University of Brasilia, Ceilândia Sul, Brasília 72220-275, DF, Brazil
9 Laboratory of Electron Paramagnetic Resonance, Institute of Physics, University of Brasília, Campus Universitário Darcy Ribeiro, Brasília 70910-900, DF, Brazil; psouza@unb.br (P.E.N.S.)
10 Department of Pathology, Faculty of Medicine, University of Brasilia, Campus Universitário Darcy Ribeiro, Brasília 70910-900, DF, Brazil; elizacarladuarte@gmail.com (E.C.B.D.)
11 Post-Graduation Program in Biomedical Engineering-PPGEB, Faculty of Gama-FGA, University of Brasilia, St. Leste Projeção A–Gama Leste, Brasília 72444-240, DF, Brazil; marbretas@unb.br (M.L.B.C.)
12 Nursing Course, School of Ceilândia, Campus Universitário—Centro Metropolitano, University of Brasilia, Ceilândia Sul, Brasília 72220-275, DF, Brazil
* Corresponding author: gjoanitti@unb.br

Citation: Pinheiro, A.C.; Ombredane, A.S.; Pinheiro, W.O.; Andrade, L.R.; Silva, V.R.P.; Felice, G.J.; Alves, D.S.; Albernaz, A.F.; Silveira, A.P.; Lima, M.C.F.; et al. Evaluation of Biocompatibility, Anti-Inflammatory, and Antinociceptive Activities of Pequi Oil-Based Nanoemulsions in In Vitro and In Vivo Models. *Nanomaterials* **2022**, *12*, 4260. https://doi.org/10.3390/nano12234260

Academic Editor: Ying Liu

Received: 1 October 2022
Accepted: 31 October 2022
Published: 30 November 2022

Publisher's Note: MDPI stays neutral with regard to jurisdictional claims in published maps and institutional affiliations.

Copyright: © 2022 by the authors. Licensee MDPI, Basel, Switzerland. This article is an open access article distributed under the terms and conditions of the Creative Commons Attribution (CC BY) license (https://creativecommons.org/licenses/by/4.0/).

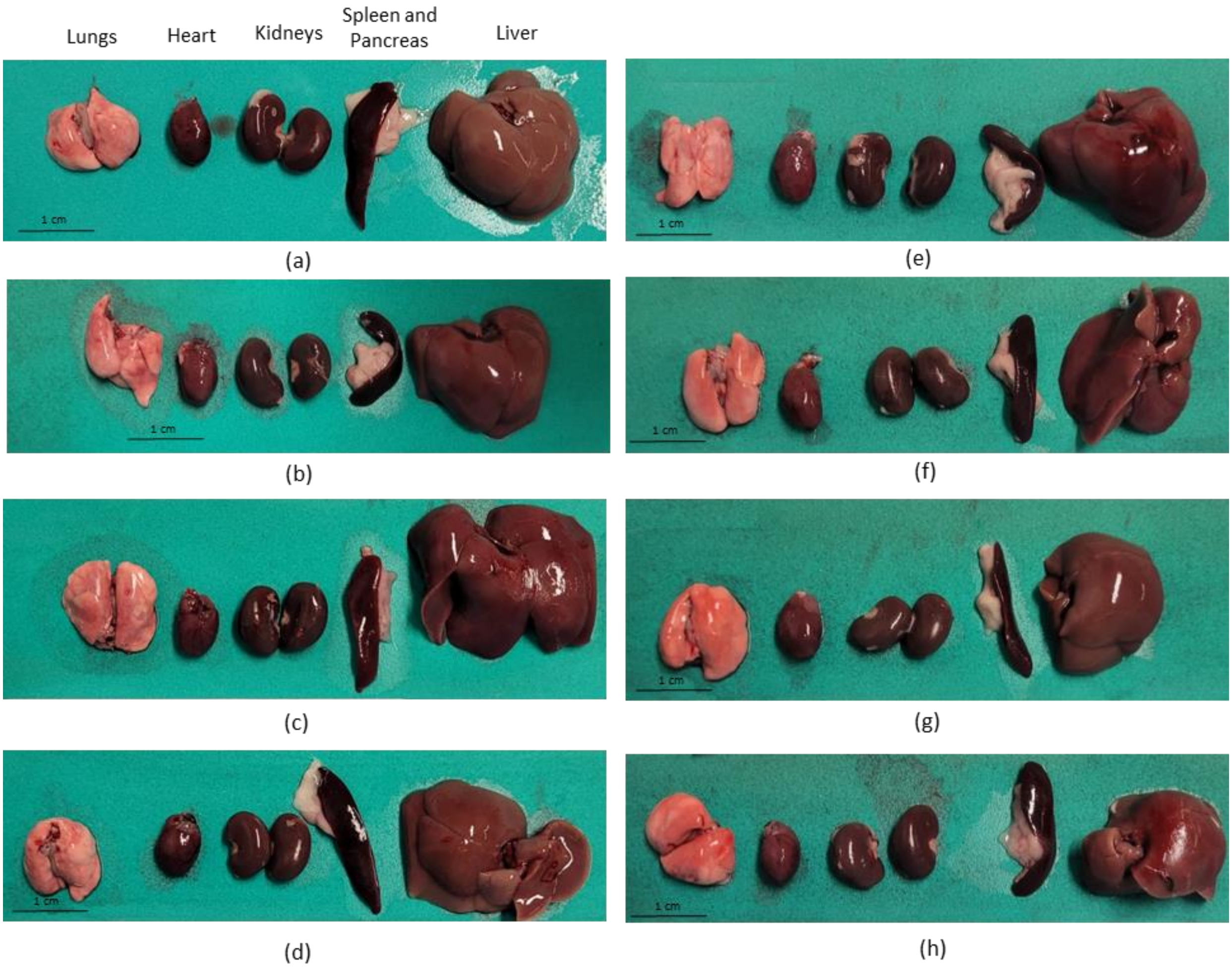

Figure S1. Macroscopic photos of the organs of a representative animal of each experimental group, containing lung, heart, kidneys, pancreas associated with the spleen, liver, respectively. (**a**) Control PBS; (**b**) Dexamethasone (2 mg/Kg/i.p.); (**c**) Free oil (100 mg/kg); (**d**) Free oil (400 mg/kg); (**e**) PeNE (100 mg/kg); (**f**) PeNE (400 m/kg); (**g**) Blank (100 mg/kg); (**h**) Blank (400 mg/kg).
